# Sensitivity analysis of the top-quark sector

**DOI:** 10.1140/epjp/s13360-026-07790-7

**Published:** 2026-05-27

**Authors:** Fernando Cornet-Gomez, Víctor Miralles, Marcos Miralles López, María Moreno Llácer, Marcel Vos

**Affiliations:** 1 Departamento de Física, Universidad de Córdoba, Campus Universitario de Rabanales, Ctra. N-IV Km. 396, 14071 Córdoba, Spain; 2 Departament de Física, Universitat d’Alacant, Campus de Sant Vicent del Raspeig, 03690 Alicante, Spain; 3 School of Physics and Astronomy, University of Glasgow, Glasgow, G12 8QQ Scotland, UK; 4 IFIC, Universitat de València and CSIC, Calle Catedrático José Beltrán 2, 46980 Paterna, Spain

## Abstract

We study the sensitivity of current and future collider observables to top-quark SMEFT operators through a one-operator-at-a-time analysis. Using data from the Tevatron, LEP, and LHC Run 2, as well as projections for the HL-LHC and future lepton colliders, we identify the measurements that provide the strongest individual constraints. This approach clarifies the role of specific observables in the top-quark SMEFT program and highlights the significant improvement in sensitivity expected at future facilities.

## Introduction

The standard model effective field theory (SMEFT) has become the primary framework for interpreting top-quark measurements at the Large Hadron Collider (LHC) and planning for future collider programs. By parameterizing potential new physics (NP) through higher-dimensional operators, the SMEFT allows for a model-independent quantification of the agreement between data and theory.

While recent global fits [1, 2] provide a comprehensive view of the current constraints on the top-quark sector [3, 4], they often mask the specific sensitivity of individual observables due to the large number of degrees of freedom and the presence of “blind directions” in the parameter space. In a global analysis, correlations between Wilson coefficients (WCs) can significantly weaken the marginalized bounds, potentially obscuring the underlying precision of the experimental measurements. Indeed, in the case in which the NP does not follow the precise correlations pattern obtained in the global fits, the marginalized bounds would underestimate the constraints.

In this report, we shift the focus from the global marginalized result to a detailed technical assessment of individual constraints. We present the 95% probability intervals for a set of 29 Wilson coefficients, obtained by varying one operator at a time while fixing all others to their standard model values. This approach serves two primary purposes. First, it identifies the “golden channels” and specific differential distributions that provide the most stringent constraints on particular operator classes. And second, it provides a baseline for experimentalists to evaluate the impact of new measurements.

We include current data from the Tevatron, LEP, and LHC Run 2, and provide projections for the High-Luminosity LHC (HL-LHC) and future lepton colliders, including $e^+e^-$ Higgs factories and the muon collider. By isolating the impact of individual observables, we demonstrate how future facilities could reach sensitivities for four-fermion operators as low as $\mathcal {O}(10^{-4})$ TeV$^{-2}$.

## Theoretical framework and methodology

The analysis is performed within the SMEFT framework, where the effective Lagrangian is expanded as:

1 $$\begin{aligned} \mathcal {L}_{\text {eff}} = \mathcal {L}_{\text {SM}} + \frac{1}{\Lambda ^2} \sum _i C_i O_i + \mathcal {O}\left( \Lambda ^{-4} \right) . \end{aligned}$$

Following the decoupling theorem [5], we focus on dimension-six operators built from SM fields, assuming *CP* conservation^1^ and a flavor symmetry $U(2)^5$ that distinguishes the third generation [7]. We employ the Warsaw basis [8] and follow the prescription of the LHC top-quark Working Group [9] for the linear combinations of Wilson coefficients (WCs).^2^

### Current observables and experimental input

The experimental foundation of this work is the dataset from the Tevatron, LEP, and LHC Run 2. This includes a wide array of top-quark processes: inclusive and differential $t\bar{t}$ production, single-top production in all channels (*t*, *s*, and *tW*), and associated production with gauge bosons ($t\bar{t}Z$, $t\bar{t}\gamma$, $t\bar{t}W$).

As detailed in Ref. [1], our analysis also incorporated measurements of the quantum entanglement in the $t\bar{t}$ pair production at threshold [10, 11] and in the boosted regime [12]. Although these measurements were part of the global baseline, their individual impact on the specific operators was already presented in Ref. [1] so it will not be discussed in this report.

### Future projections

To provide a roadmap for the top-quark sector, we include projections for the High-Luminosity LHC (HL-LHC), as well as future lepton collider. These include $e^+e^-$ Higgs factories (ILC and FCC-ee) and a high-energy muon collider operating at the 3–30 TeV range. The detailed simulation parameters for these future scenarios are provided in Ref. [1].

### Simulation and fit procedure

In this technical report, we made public the systematic mapping of individual WC sensitivities for each observable. While the global fits in Ref. [1] provide the most statistically rigorous bounds by allowing all operators to vary simultaneously, they inevitably introduce correlations that can mask the inherent precision of a measurement.

In the following section, we present the limits obtained including a single observable and by varying a single WC at a time. This sensitivity analysis allows for the identification of the most powerful observables for each operator, providing a clear diagnostic tool for understanding the current and future landscape of the top-quark SMEFT fit.

In general, the physical observables contain a linear dependence on the WCs coming from the interference of the pure dimension-six operators with the SM and a quadratic contribution from squaring dimension-six operators. In this report, we strictly adopt the linear truncation, including only terms proportional to $\Lambda ^{-2}$. This approach ensures a conservative and theoretically consistent treatment, as a full $\mathcal {O}(\Lambda ^{-4})$ analysis would require the inclusion of dimension-eight operator interferences [13].

The linear coefficients $X^{\mathrm{{int}}}_i$ are obtained using MadGraph5_aMC@NLO [14] with the SMEFTsim [15] and SMEFT@NLO [16] models. Statistical inference is carried out via a Bayesian global fit implemented in the HEPfit [17] package.

## Results: individual sensitivity breakdown

**Fig. 1:**
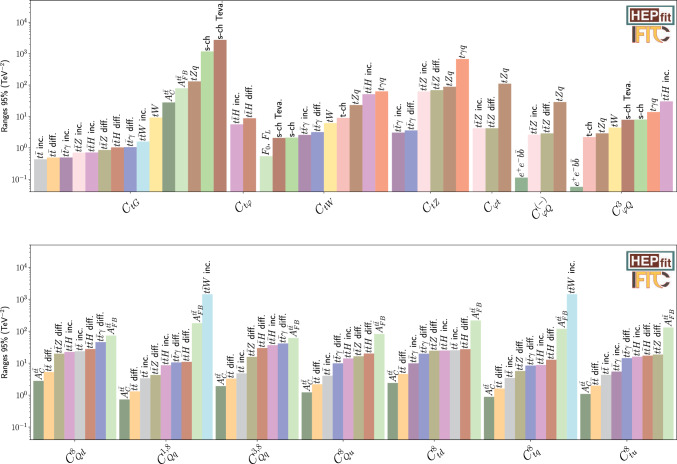
Comparison of the individual 95% probability bounds for the two-quark (top) and four-quark operators octets (bottom), derived from the different measurements at current colliders (LHC, LEP and Tevatron). The individual bounds are obtained from fits of a single operator coefficient to a single measurement. Similar results are obtained for the singlet coefficients

In this section, we break down the 95% probability intervals according to the experimental facility providing the constraint. By isolating the impact of each observable for the different future colliders, we demonstrate how the top-quark SMEFT landscape could evolve depending on which machine the community decides to build.

### Current constraints and HL-LHC projections

**Fig. 2:**
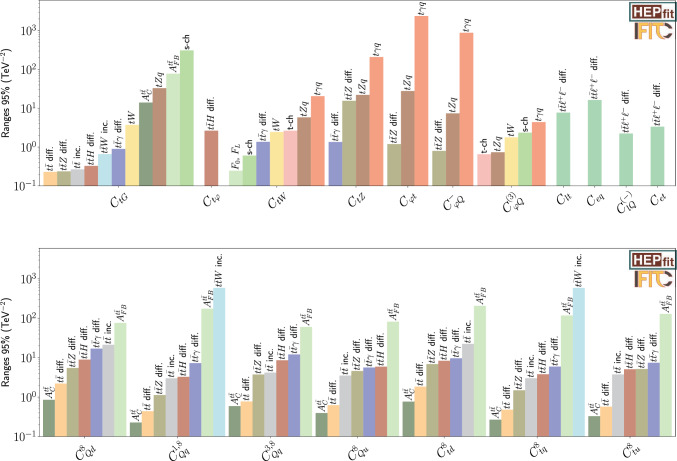
Comparison of the individual 95% probability bounds for the two-quark (top) and four-quark operators octets (bottom), derived from the different measurements at the HL-LHC. The individual bounds are obtained from fits of a single operator coefficient to a single measurement. Similar results are obtained for the singlet coefficients

As discussed, current experimental bounds rely on the Tevatron, LEP, and LHC Run 2 datasets. The individual sensitivities can be found in Fig. 1. The four-quark sector is predominantly constrained by the $t\bar{t}$ charge asymmetry and the differential $t\bar{t}$ distribution at the LHC. The landscape at the HL-LHC (see Fig. 2) is similar but with an overall improvement of a factor of roughly 3. For some of the two-quark operators, like $C_{\varphi Q}^{(-)}$ and $C_{\varphi Q}^{(3)}$, the most constraining observable comes from LEP. Although HL-LHC will improve LHC bounds, it will not be able to surpass the one that comes from the $e^+e^-\rightarrow b\bar{b}$ observable. The HL-LHC is also expected to give bounds to two-quark–two-lepton operators as $C_{lt}, C_{eq}, C_{lq}^{(-)}$ and $C_{et}$

### Future lepton and muon colliders

**Fig. 3:**
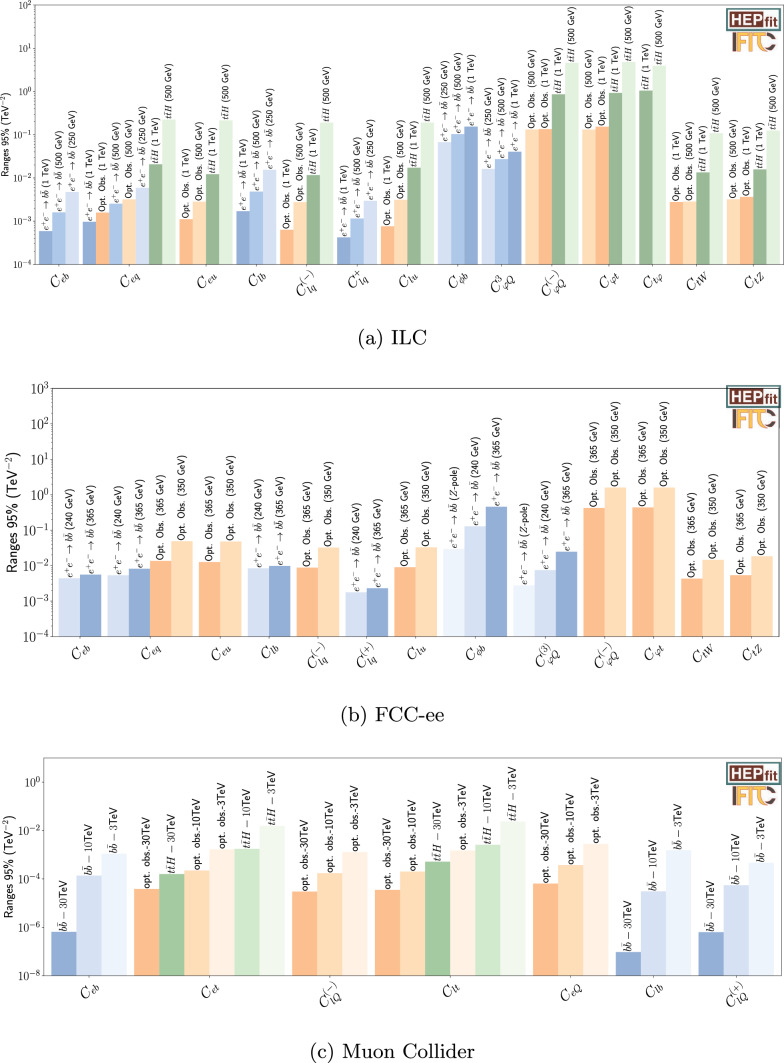
Comparison of the individual 95% probability bounds derived from different measurements at future lepton colliders: **a** ILC, **b** FCC-ee, and **c** muon collider. The individual bounds are obtained from fits of a single operator coefficient to a single measurement. For the muon collider case, the two-fermion coefficients can be found in Ref. [1]

Future facilities offer a clean environment to reach sensitivities inaccessible to hadronic machines. The $e^+e^-$ Higgs factories (ILC/FCC-ee) are essential for constraining the two-quark–two-lepton sector, as shown in Fig. 3a and b. Our results show that they can achieve enough sensitivities to constrain some operators up to $\mathcal {O}(10^{-4})$ TeV$^{-2}$ like $C_{lu}$ and $C_{eq}$.

There have also been interest in the community for a possible future muon collider. The main advantage is the possibility of operating at much higher energies. Indeed, a muon collider operating in the 3–30 TeV range represents the ultimate frontier for vertex corrections (${C}_{tG}$, $C_{\varphi Q}^{(-)}$) and dipole terms as can be shown in Fig. 3c.

## Footnotes

1 Some of the *CP* violating interactions have also been studied in Ref. [6].

2 The full list of the WC considered can be found in Ref. [1]

## References

[1] F. Cornet-Gomez, V. Miralles, M. Miralles López, M. Moreno Llácer, M. Vos, Future collider constraints on top-quark operators. JHEP **10**, 156 (2025). 10.1007/JHEP10(2025)156. arXiv:2503.11518 [hep-ph]

[2] J. Blas, A. Goncalves, V. Miralles, L. Reina, L. Silvestrini, M. Valli, Constraining new physics effective interactions via a global fit of electroweak, Drell-Yan, Higgs, top, and flavour observables. JHEP **03**, 013 (2026). 10.1007/JHEP03(2026)013. arXiv:2507.06191 [hep-ph]

[3] G. Durieux, A. Irles, V. Miralles, A. Peñuelas, R. Pöschl, M. Perelló, M. Vos, The electro-weak couplings of the top and bottom quarks–global fit and future prospects. JHEP **12**, 98 (2019). 10.1007/JHEP12(2019)098. arXiv:1907.10619 [hep-ph]. [Erratum: JHEP 01, 195 (2021)]

[4] V. Miralles, M.M. López, M.M. Llácer, A. Peñuelas, M. Perelló, M. Vos, The top quark electro-weak couplings after LHC Run 2. JHEP **02**, 032 (2022). 10.1007/JHEP02(2022)032. arXiv:2107.13917 [hep-ph]

[5] T. Appelquist, J. Carazzone, Infrared singularities and massive fields. Phys. Rev. D **11**, 2856 (1975). 10.1103/PhysRevD.11.2856

[6] V. Miralles, Y. Peters, E. Vryonidou, J.K. Winter, Sensitivity to -violating effective couplings in the top-Higgs sector (2024) arXiv:2412.10309 [hep-ph]

[7] D.A. Faroughy, G. Isidori, F. Wilsch, K. Yamamoto, Flavour symmetries in the SMEFT. JHEP **08**, 166 (2020). 10.1007/JHEP08(2020)166. arXiv:2005.05366 [hep-ph]

[8] B. Grzadkowski, M. Iskrzynski, M. Misiak, J. Rosiek, Dimension-six terms in the standard model Lagrangian. JHEP **10**, 085 (2010). 10.1007/JHEP10(2010)085. arXiv:1008.4884 [hep-ph]

[9] J.A. Aguilar-Saavedra, C. Degrande, G. Durieux, F. Maltoni, E. Vryonidou, C. Zhang, Interpreting top-quark LHC measurements in the standard-model effective field theory. arXiv:1802.07237 (hep-ph)

[10] The ATLAS collaboration, Observation of quantum entanglement with top quarks at the ATLAS detector. Nature **633**(8030), 542–547 (2024). 10.1038/s41586-024-07824-z. arXiv:2311.07288 [hep-ex] 39294352 10.1038/s41586-024-07824-z PMC11410654

[11] The CMS collaboration, Observation of quantum entanglement in top quark pair production in proton–proton collisions at TeV. Rept. Prog. Phys. **87**(11), 117801 (2024). 10.1088/1361-6633/ad7e4d. arXiv:2406.03976 [hep-ex] 10.1088/1361-6633/ad7e4d 39315475

[12] The CMS collaboration, Measurements of polarization and spin correlation and observation of entanglement in top quark pairs using lepton+jets events from proton-proton collisions at s=13 TeV. Phys. Rev. D **110**(11), 112016 (2024). 10.1103/PhysRevD.110.112016. arXiv:2409.11067 [hep-ex]

[13] I. Brivio, Truncation, validity, uncertainties. arXiv:2201.04974 (hep-ph) (2022)

[14] J. Alwall, R. Frederix, S. Frixione, V. Hirschi, F. Maltoni, O. Mattelaer, H.-S. Shao, T. Stelzer, P. Torrielli, M. Zaro, The automated computation of tree-level and next-to-leading order differential cross sections, and their matching to parton shower simulations. JHEP **07**, 079 (2014). 10.1007/JHEP07(2014)079. arXiv:1405.0301 [hep-ph]

[15] I. Brivio, SMEFTsim 3.0—a practical guide. JHEP **04**, 073 (2021). 10.1007/JHEP04(2021)073. arXiv:2012.11343 [hep-ph]

[16] C. Degrande, G. Durieux, F. Maltoni, K. Mimasu, E. Vryonidou, C. Zhang, Automated one-loop computations in the standard model effective field theory. Phys. Rev. D **103**(9), 096024 (2021). 10.1103/PhysRevD.103.096024. arXiv:2008.11743 [hep-ph]

[17] J. De Blas et al., HEPfit: a code for the combination of indirect and direct constraints on high energy physics models. Eur. Phys. J. C **80**(5), 456 (2020). 10.1140/epjc/s10052-020-7904-z. arXiv:1910.14012 [hep-ph]

